# Supplementary material for: Immunogenicity of the 13-Valent Pneumococcal Conjugate Vaccine (PCV13) Followed by the 23-Valent Pneumococcal Polysaccharide Vaccine (PPSV23) in Adults with and without Immunosuppressive Therapy
Source: Vaccines (Basel). 2022 May 17;10(5):795. doi: 10.3390/vaccines10050795 (PMC9146363; doi:10.3390/vaccines10050795)
Supplement: Supplementary file 1 [file vaccines-10-00795-s001.zip › vaccines-1711688-supplementary.pdf]

Supplementary materials

**Table S1. Seroprotection rates excluding anti-CD20 therapy (sensitivity analysis)**					
**A. All 24 serotypes**	**T0[1]**	**T2**	**T4**	**T6**	**T12**
Controls	1/36 (2.8)	10/32 (31)	28/34 (82)[a]	24/32 (75)[a]	22/35 (63)[a]
cIM[2]	0/47 (0)	5/44 (11)	26/45 (58)	21/39 (54)	21/44 (48)
bIM[3]	0/44 (0)	6/43 (14)	26/41 (63)	19/35 (54)	18/40 (45)
Combination	0/58 (0)	5/55 (8.8)	23/53 (43)[b]	17/49 (35)[b]	14/56 (25)[b]
Switched	0/21 (0)	5/21 (24)	12/20 (60)	10/19 (53)	7/17 (41)
p-value across groups	0.31	0.05	**0.01**	**0.01**	**0.01**
**B. PCV13[4] serotypes**	**T0**	**T2**	**T4**	**T6**	**T12**
Controls	0/36 (0)	24/32 (75)[a]	29/34 (85)[a]	24/32 (75)[a]	20/35 (57)[a]
cIM	2/47 (4.3)	25/44 (57)	24/45 (53)[b]	21/39 (54)	18/44 (41)
bIM	0/44 (0)	26/43 (61)	24/41 (59)	19/35 (54)	16/40 (40)
Combination	1/58 (1.7)	22/55 (40)[b]	24/53 (45)[b]	17/49 (35)[b]	13/56 (24)[b]
Switched	0/21 (0)	15/21 (71)	12/20 (60)	11/19 (58)	7/17 (41)
p-value across groups	0.39	**0.01**	**0.01**	**0.01**	**0.03**
**C. PPSV23[5]-exclusive**	**T0**	**T2**	**T4**	**T6**	**T12**
Controls	1/ 36 (2.8)	3/32 (9.4)	28/34 (82)	27/32 (84)[a]	26/35 (74)[a]
cIM	0/47 (0)	0/44 (0)	29/45 (64)	21/39 (54)	23/44 (52)
bIM	1/44 (2.3)	1/43 (2.3)	30/41 (73)	24/35 (69)	22/40 (55)
Combination	1/58 (1.7)	2/55 (3.6)	28/53 (53)	26/49 (53)[b]	23/56 (41)[b]
Switched	0/21 (0)	1/21 (4.8)	10/20 (50)	7/19 (37)[b]	6/17 (35)
p-value across groups	0.80	0.29	0.03	**0.01**	**0.02**

Table S2: Serotype specific seroprotection rates for each time point and results of generalized linear mixed model including time point, use of immunosuppressive medication at baseline and the interaction term of the two variables. If there was no interaction the interaction term was removed.

Serotype	Time point	% >1.3 Controls	% >1.3 cIM	% >1.3 bIM	% >1.3 Combination	% >1.3 Switched	p-value medication group +/- GLMM	p-value time GLMM	Interaction timepoint* Medication + p-value if applicable
	T0	8.3	2.1	10	5.0	9.5			
	T2	63	52	57	58	57	0.95	**<0.01**	NA
1	T4	59	60	54	61	55			
	T6	50	59	54	53	47			
	T12	46	48	46	40	35			
	T0	47	26	36	37	38			
	T2	53	34	43	44	48	0.24	**<0.01**	NA
2	T4	97	87	91	93	90			
	T6	97	82	92	92	90			
	T12	94	82	85	79	94			
	T0	8.3	4.3	2.0	3.3	19	0.08	**<0.01**	NA
3	T2	34	27	14	12	19			
	T4	32	33	20	15	15			

Table S2: Serotype specific seroprotection rates for each time point and results of generalized linear mixed model including time point, use of immunosuppressive medication at baseline and the interaction term of the two variables. If there was no interaction the interaction term was removed.

Serotype	Time point	% >1.3 Controls	% >1.3 cIM	% >1.3 bIM	% >1.3 Combination	% >1.3 Switched	p-value medication group +/- GLMM	p-value time GLMM	Interaction timepoint* Medication + p-value if applicable
	T6	19	31	10	7.8	21			
	T12	11	18	11	5.2	5.9			
4	T0	2.8	2.8	2.1	0	6.7	**<0.01**	**<0.01**	NA
	T2	59	46	35	39	76			
	T4	77	47	33	39	65			
	T6	40	39	28	39	58			
	T12	46	32	22	26	29			
5	T0	11	6.4	6.0	3.3	9.5	**<0.01**	**<0.01**	NA
	T2	78	59	49	53	43			
	T4	85	53	48	48	55			
	T6	78	49	46	51	42			
	T12	63	46	35	29	35			
6A	T0	28	13	18	20	9.5	0.16	**<0.01**	NA
	T2	84	66	71	70	81			
	T4	88	58	67	70	80			
	T6	78	59	62	67	74			
	T12	71	52	57	53	71			
6B	T0	11	13	18	15	14	0.84	**<0.01**	NA
	T2	66	57	63	65	81			
	T4	77	60	65	63	70			
	T6	72	62	54	59	63			
	T12	60	55	48	52	53			
7F	T0	25	21	28	28	24	0.72	**<0.01**	NA
	T2	91	68	80	77	81			
	T4	97	73	85	83	80			
	T6	88	69	74	73	74			
	T12	77	66	65	62	65			
8	T0	36	28	34	45	43	0.36	**<0.01**	NA
	T2	41	27	43	47	52			
	T4	94	78	85	89	90			
	T6	97	74	85	80	90			
	T12	89	75	74	74	82			
9N	T0	17	15	18	25	19	0.60	**<0.01**	NA
	T2	53	36	34	35	33			
	T4	88	73	65	70	65			
	T6	88	74	64	61	68			
	T12	71	64	57	55	53			
9V	T0	11	13	8	18	19	**0.03**	**<0.01**	0.02
	T2	88	59	57	53	68			
	T4	88	58	61	56	55			
	T6	84	54	54	41	53			
	T12	71	48	39	31	47			
10A	T0	36	26	22	20	14	0.16	**<0.01**	NA
	T2	41	32	27	21	19			
	T4	68	69	63	61	50			

Table S2: Serotype specific seroprotection rates for each time point and results of generalized linear mixed model including time point, use of immunosuppressive medication at baseline and the interaction term of the two variables. If there was no interaction the interaction term was removed.

Serotype	Time point	% >1.3 Controls	% >1.3 cIM	% >1.3 bIM	% >1.3 Combination	% >1.3 Switched	p-value medication group +/- GLMM	p-value time GLMM	Interaction timepoint* Medication + p-value if applicable
	T6	66	64	54	67	42			
	T12	63	68	52	52	35			
11A	T0	22	26	24	35	38	0.69	**<0.01**	0.01
	T2	31	21	27	37	38			
	T4	82	64	67	61	75			
	T6	75	64	64	65	79			
	T12	77	66	59	50	59			
12F	T0	5.6	0	4	1.7	0	**0.02**	**<0.01**	NA
	T2	9.4	0	6.1	3.5	4.8			
	T4	41	44	28	22	35			
	T6	44	36	28	16	26			
	T12	31	32	24	16	18			
14	T0	39	55	40	47	29	0.24	**<0.01**	NA
	T2	84	91	82	83	96			
	T4	85	96	83	82	95			
	T6	81	92	82	78	95			
	T12	83	91	76	74	77			
15B	T0	38	45	40	25	29	0.18	**<0.01**	NA
	T2	56	52	47	42	38			
	T4	91	84	80	82	85			
	T6	93	82	77	80	74			
	T12	91	84	76	71	65			
17F	T0	28	26	26	23	33	0.60	**<0.01**	NA
	T2	25	30	29	28	43			
	T4	88	79	74	70	75			
	T6	88	80	74	67	63			
	T12	80	71	59	57	47			
18C	T0	22	21	16	23	24	0.29	**<0.01**	NA
	T2	91	75	76	67	76			
	T4	97	80	70	69	70			
	T6	91	77	69	65	68			
	T12	86	68	70	57	59			
19A	T0	69	32	36	42	48	**0.01**	**<0.01**	NA
	T2	100	84	86	88	86			
	T4	97	80	83	87	85			
	T6	97	80	80	84	95			
	T12	89	77	70	76	82			
19F	T0	39	30	22	35	19	0.15	**<0.01**	NA
	T2	88	80	78	79	91			
	T4	94	80	78	80	90			
	T6	94	77	77	75	90			
	T12	89	73	65	64	77			
20	T0	53	23	50	47	33	**0.02**	**<0.01**	NA
	T2	56	32	61	47	43			
	T4	91	80	85	82	65			

Serotype	Time point	% >1.3 Controls	% >1.3 cIM	% >1.3 bIM	% >1.3 Combination	% >1.3 Switched	p-value medication group +/- GLMM	p-value time GLMM	Interaction timepoint* Medication + p-value if applicable
	T6	88	77	85	73	74			
	T12	91	71	78	71	59			
22F	T0	5.6	8.5	14	12	5	0.07	**<0.01**	NA
	T2	13	4.5	12	12	9.5			
	T4	77	58	67	50	60			
	T6	69	56	64	49	47			
	T12	63	55	52	36	41			
23F	T0	25	11	8.0	22	19	**0.01**	**<0.01**	NA
	T2	84	68	63	63	91			
	T4	88	64	61	65	85			
	T6	84	59	51	61	95			
	T12	80	50	46	48	65			
33F	T0	50	43	44	37	24	0.12	**<0.01**	NA
	T2	47	41	45	35	33			
	T4	85	89	83	72	90			
	T6	88	92	82	71	95			
	T12	89	93	80	66	88			
12A*	T0	47	36	26	25	24	0.12	**<0.01**	NA
	T2	56	39	37	25	29			
	T4	56	47	44	30	25			
	T6	53	39	41	29	31			
	T12	43	39	38	29	37			
45*	T0	89	77	78	78	76	0.62	0.80	NA
	T2	94	80	78	79	76			
	T4	91	78	83	82	75			
	T6	91	82	85	80	79			
	T12	86	84	87	78	77			

cIM= conventional immunomodulator, bIM = biological immunomodulator, combi = combination therapy

* 12A and 45 serotypes are in AU/ml, the cut-off has no actual meaning except for statistical comparison between groups and time-points.

T= time point; Blue = PCV13 serotypes, white = PPSV23-Non-PCV13 serotypes, green= non-vaccine serotypes

Bold highlights statlistically significant outcme

Table S3 Factors associated with seroprotection (all serotypes) over time (multivariable generalized linear mixed model)				
**Model Term**	p-value	Odds ratio	95% Confidence Interval for Exp(Coefficient)	
			Lower	Upper
**Age**	0.08	0.99	0.97	1.00
**Sex**	0.729	1.082	0.691	1.697
**T12**	<0.01	59	17	210
**T6**	<0.01	95	27	333
**T4**	<0.01	124	37	421
**T2**	<0.01	13	4.4	41
**T0**	ref			
**Switched**	0.044	0.40	0.16	0.98
**Combi**	<0.01	0.18	0.09	0.36
**bIM**	<0.01	0.33	0.16	0.66
**cIM**	0.010	0.40	0.20	0.82
**Control**	ref			

Table S4 Geometric mean concentrations and geometric mean fold rises compared to baseline.

		**T0**			**T2**						**T4**						**T6**						**T12**					
			95% CI			95% CI			95% CI			95% CI			95% CI			95% CI			95% CI			95% CI			95% CI	
**Serotype**	**Group**	**GMC**	Lower	Upper	GMC	Lower	Upper	GMFR	Lower	Upper	GMC	Lower	Upper	GMFR	Lower	Upper	GMC	Lower	Upper	GMFR	Lower	Upper	GMC	Lower	Upper	GMFR	Lower	Upper
1	controls	0.13	0.08	0.21	2.5	1.3	4.6	**16**	**10**	**27**	2.3	1.3	4.2	**19**	**12**	**31**	1.6	0.88	2.8	**13**	**8.4**	**21**	1.3	0.74	2.2	**10**	**6.9**	**16**
	cIM	0.14	0.09	0.21	1.6	0.93	2.7	**12**	**7.7**	**18**	2.0	1.2	3.2	**14**	**9.6**	**21**	1.6	0.96	2.8	**12**	**7.6**	**18**	1.2	0.74	2.0	**8.3**	**5.8**	**12**
	bIM	0.15	0.10	0.23	1.8	1.1	3.1	**13**	**8.5**	**19**	2.4	1.4	3.9	**15**	**10**	**22**	1.6	0.96	2.8	**11**	**7.5**	**17**	1.2	0.72	1.9	**7.8**	**5.5**	**11**
	Combi	0.13	0.09	0.20	1.5	0.96	2.5	**12**	**8.1**	**17**	1.9	1.2	3.0	**13**	**9.2**	**19**	1.4	0.88	2.2	**11**	**7.4**	**15**	0.81	0.52	1.2	**5.9**	**4.3**	**8.2**
	Switched	0.11	0.06	0.21	1.7	0.80	3.8	**16**	**8.8**	**30**	1.6	0.76	3.5	**17**	**9.6**	**32**	1.2	0.56	2.6	**10**	**5.7**	**19**	0.69	0.31	1.5	**7.4**	**4.1**	**13**
2	controls	1.4	0.84	2.3	1.6	0.93	2.8	1.2	0.94	1.5	27	16	47	**20**	**11**	**35**	25	15	43	**18**	**11**	**30**	14	8.0	23	**10**	**6.1**	**16.8**

Table S4 Geometric mean concentrations and geometric mean fold rises compared to baseline.																												
		T0			T2						T4						T6						T12					
			95% CI			95% CI			95% CI			95% CI			95% CI			95% CI			95% CI			95% CI			95% CI	
**Serotype**	**Group**	**GMC**	Lower	Upper	GMC	Lower	Upper	GMFR	Lower	Upper	GMC	Lower	Upper	GMFR	Lower	Upper	GMC	Lower	Upper	GMFR	Lower	Upper	GMC	Lower	Upper	GMFR	Lower	Upper
	cIM	0.56	0.36	0.87	0.64	0.40	1.0	1.1	0.90	1.4	10	6.4	17	17	10	27	7.5	4.6	12	16	9.7	25	5.9	3.7	9.6	12	7.4	18
	bIM	0.69	0.45	1.0	0.82	0.53	1.3	1.2	0.98	1.5	12	7.2	18	15	9.2	24	10	6.4	17	14	8.8	23	6.4	4.0	10	9.2	5.9	14
	Combi	0.91	0.62	1.3	1.10	0.73	1.7	1.2	0.98	1.4	8.7	5.7	13	9.8	6.3	15	6.9	4.5	11	7.8	5.1	12	4.8	3.1	7.24	5.2	3.5	7.8
	Switched	0.70	0.37	1.3	1.2	0.60	2.3	1.7	1.2	2.3	21	10	43	32	16	67	14	6.9	28	20	10	39	9.3	4.3	20	12	5.6	24
3	controls	0.11	0.07	0.18	0.52	0.32	0.84	4.6	3.2	6.5	0.75	0.48	1.2	6.4	4.3	9.5	0.46	0.29	0.73	4.2	3.0	6.0	0.33	0.21	0.51	3.0	2.2	4.1
	cIM	0.11	0.08	0.17	0.44	0.29	0.66	3.8	2.8	5.2	0.55	0.37	0.82	5.0	3.5	7.0	0.45	0.30	0.69	3.9	2.8	5.4	0.33	0.23	0.50	2.8	2.1	3.7
	bIM	0.10	0.07	0.16	0.34	0.23	0.50	3.2	2.4	4.2	0.44	0.30	0.65	3.9	2.8	5.5	0.34	0.22	0.51	3.0	2.2	4.1	0.27	0.19	0.40	2.4	1.8	3.1
	Combi	0.07	0.05	0.11	0.28	0.19	0.40	4.1	3.1	5.3	0.34	0.24	0.49	5.0	3.6	6.8	0.25	0.17	0.36	3.4	2.6	4.5	0.18	0.13	0.25	2.4	1.9	3.0
	Switched	0.10	0.05	0.18	0.54	0.30	0.98	5.6	3.6	8.7	0.53	0.29	0.94	5.3	3.2	8.9	0.43	0.23	0.78	4.0	2.5	6.4	0.23	0.12	0.44	2.5	1.6	3.9
4	controls	0.12	0.08	0.18	1.6	0.89	2.8	11	6.5	19	2.1	1.3	3.6	17	10	29	1.3	0.78	2.2	11	6.5	18	0.89	0.54	1.46	7.7	4.9	12
	cIM	0.11	0.07	0.15	1.0	0.64	1.7	9.5	6.0	15	1.1	0.73	1.8	11	7.2	17	0.81	0.50	1.3	8.3	5.3	13	0.66	0.42	1.02	5.8	3.9	8.6
	bIM	0.08	0.06	0.12	0.91	0.57	1.4	11	7.3	17	0.80	0.51	1.2	9.7	6.3	15	0.62	0.39	1.00	7.8	4.9	12	0.44	0.29	0.68	5.7	3.8	8.4
	Combi	0.13	0.09	0.18	1.1	0.71	1.7	8.2	5.5	12	1.1	0.73	1.7	8.4	5.6	13	0.81	0.54	1.2	6.2	4.2	9.3	0.56	0.38	0.82	4.2	3.0	5.9
	Switched	0.11	0.06	0.19	3.2	1.6	6.6	29	15	57	2.1	1.1	4.1	21	11	40	1.6	0.82	3.2	13	6.8	25	0.72	0.35	1.5	9.3	4.9	18
5	controls	0.26	0.17	0.39	4.6	2.3	9.0	16	9.0	27	5.1	2.79	9.18	20	13	33	3.9	2.2	7.2	15	9.1	23	2.2	1.3	3.9	8.9	5.8	14
	cIM	0.16	0.11	0.24	1.6	0.90	2.8	8.7	5.5	14	1.8	1.07	3.01	11	7.4	17	1.6	0.96	2.8	9.6	6.3	15	1.3	0.78	2.1	7.5	5.1	10.9
	bIM	0.15	0.11	0.22	1.2	0.68	2.0	7.8	5.0	12	1.4	0.82	2.3	9.0	6.0	14	1.4	0.82	2.4	8.5	5.6	13	0.89	0.56	1.4	5.6	3.8	8.0
	Combi	0.12	0.09	0.17	1.2	0.72	2.0	9.6	6.4	14	1.4	0.87	2.23	11	7.8	17	1.2	0.77	2.0	9.3	6.4	14	0.65	0.43	1.0	5.3	3.8	7.4
	Switched	0.15	0.09	0.26	1.7	0.72	3.8	11	5.7	22	1.6	0.72	3.42	11	5.8	20	1.0	0.47	2.2	7.8	4.3	14	0.83	0.38	1.8	5.1	2.8	9.4
6A	controls	0.38	0.22	0.66	8.6	4.4	17	22	13	37	7.5	3.9	14	19.1	12	32	5.5	2.92	10	15	9.21	26	3.7	2.0	6.6	9.5	5.9	15
	cIM	0.15	0.10	0.25	2.5	1.4	4.4	15	9.7	24	2.2	1.2	3.8	14	9.2	22	1.8	1.03	3.2	13	8.05	20	1.5	0.90	2.6	8.8	5.7	13
	bIM	0.24	0.15	0.39	3.4	2.0	5.8	13	8.6	20	2.0	1.2	3.5	8.5	5.5	13	1.4	0.80	2.5	6.75	4.26	11	1.3	0.79	2.2	5.5	3.6	8.4
	Combi	0.29	0.19	0.44	3.1	1.9	5.1	11	7.2	16	2.5	1.5	4.2	9.4	6.3	14	2.3	1.40	3.8	7.56	5.05	11	1.3	0.83	2.1	4.6	3.2	6.7
	Switched	0.21	0.10	0.42	6.9	3.0	16	33	17	64	5.1	2.2	12	26	13	50	3.8	1.69	8.6	17	8.98	34	2.9	1.2	6.7	14	6.9	27
6B	controls	0.26	0.15	0.45	5.0	2.4	10	21	12	37	6.0	3.1	12	21	13	35	3.7	1.91	7.1	15	9.3	25	2.6	1.4	4.8	9.7	6.2	15
	cIM	0.15	0.10	0.25	2.1	1.1	3.9	13	7.9	21	2.0	1.1	3.6	14	9.0	22	1.7	0.94	3.1	11	7.2	18	1.4	0.80	2.4	8.3	5.5	12
	bIM	0.18	0.11	0.28	2.2	1.2	4.0	12	7.8	19	1.9	1.1	3.4	11	6.8	16	1.3	0.73	2.4	7.8	5.0	12	1.0	0.59	1.7	5.6	3.8	8.3
	Combi	0.21	0.14	0.33	2.4	1.4	4.2	11	7.2	17	2.3	1.4	3.9	11	7.4	17	1.9	1.13	3.2	8.4	5.7	13	1.1	0.71	1.8	5.3	3.7	7.5

Table S4 Geometric mean concentrations and geometric mean fold rises compared to baseline.

		T0			T2						T4						T6						T12					
			95% CI		GMC	95% CI			95% CI		GMC	95% CI			95% CI		GMC	95% CI			95% CI		GMC	95% CI			95% CI	
**Serotype**	**Group**	**GMC**	Lower	Upper		Lower	Upper	GMFR	Lower	Upper		Lower	Upper	GMFR	Lower	Upper		Lower	Upper	GMFR	Lower	Upper		Lower	Upper	GMFR	Lower	Upper
	Switched	0.14	0.07	0.28	4.4	1.7	11	**31**	**16**	**63**	3.1	1.3	7.4	**24**	**12**	**46**	2.2	0.93	5.2	**16**	**8.4**	**31**	1.6	0.67	4.0	**11**	**5.5**	**20**
7F	controls	0.40	0.24	0.67	8.1	4.7	14	**17**	**10**	**29**	7.5	4.7	12	**20**	**13**	**32**	5.8	3.6	9.6	**13**	**8.2**	**19**	3.6	2.3	5.8	**9.0**	**6.1**	**13**
	cIM	0.46	0.30	0.72	3.2	2.0	5.0	**7.1**	**4.5**	**11**	3.7	2.5	5.66	**8.0**	**5.4**	**12**	3.0	1.9	4.7	**7.0**	**4.8**	**10**	2.1	1.4	3.2	**4.7**	**3.3**	**6.6**
	bIM	0.50	0.33	0.77	5.2	3.4	8.0	**10**	**6.6**	**16**	5.2	3.5	7.84	**11**	**7.3**	**16**	4.1	2.6	6.4	**8.0**	**5.4**	**12**	2.8	1.9	4.2	**4.9**	**3.5**	**6.9**
	Combi	0.44	0.30	0.65	3.9	2.6	5.9	**9.1**	**6.1**	**13**	4.1	2.8	5.94	**9.5**	**6.7**	**13**	3.1	2.1	4.5	**7.0**	**4.9**	**9.81**	1.8	1.2	2.5	**4.0**	**3.0**	**5.5**
	Switched	0.45	0.23	0.87	6.0	3.1	12	**13**	**7.0**	**26**	4.5	2.4	8.39	**11**	**6.2**	**19**	3.9	2.0	7.4	**9.1**	**5.2**	**16**	2.1	1.1	4.1	**5.5**	**3.2**	**9.7**
8	controls	1.2	0.73	1.8	1.4	0.84	2.3	**1.3**	**1.1**	**1.5**	14	8.4	24	**12**	**7.3**	**19**	11	6.8	19	**10**	**6.6**	**17**	7.9	4.9	13	**6.8**	**4.4**	**10**
	cIM	0.62	0.41	0.93	0.72	0.47	1.1	1.1	0.93	1.3	4.7	3.0	7.3	**7.15**	**4.7**	**11**	4.2	2.7	6.7	**5.8**	**3.8**	**8.8**	3.3	2.2	5.1	**4.9**	**3.3**	**7.1**
	bIM	0.88	0.59	1.3	1.0	0.68	1.5	1.1	1.00	1.3	6.4	4.1	10	**7.13**	**4.7**	**11**	5.4	3.4	8.6	**7.0**	**4.6**	**11**	3.4	2.2	5.2	**4.3**	**3.0**	**6.3**
	Combi	1.2	0.84	1.7	1.3	0.90	1.9	1.1	0.97	1.3	7.3	4.8	11	**6.00**	**4.1**	**8.8**	6.1	4.1	9.1	**4.6**	**3.2**	**6.6**	4.0	2.7	5.7	**3.4**	**2.4**	**4.7**
	Switched	1.2	0.64	2.1	1.7	0.89	3.1	**1.4**	**1.13**	**1.8**	11	5.8	22	**10**	**5.5**	**20**	7.0	3.6	13	**6.6**	**3.6**	**12**	3.7	1.9	7.4	**3.8**	**2.0**	**7.0**
9N	controls	0.31	0.18	0.51	1.4	0.73	2.5	**3.3**	**2.3**	**4.9**	5.6	3.2	9.7	**19**	**12**	**31**	4.6	2.6	8.1	**16**	**9.5**	**25**	3.1	1.8	5.2	**9.7**	**6.2**	**15**
	cIM	0.24	0.16	0.38	0.75	0.44	1.3	**3.0**	**2.1**	**4.1**	3.5	2.1	5.6	**15**	**9.7**	**23**	3.0	1.8	5.1	**11**	**7.0**	**17**	2.4	1.5	3.9	**9.2**	**6.2**	**14**
	bIM	0.28	0.18	0.44	0.64	0.39	1.1	**2.3**	**1.7**	**3.1**	3.1	1.9	5.0	**9.8**	**6.4**	**15**	2.3	1.4	3.8	**7.5**	**4.8**	**12**	1.5	0.91	2.3	**5.2**	**3.5**	**7.7**
	Combi	0.30	0.20	0.45	0.59	0.37	0.93	**1.9**	**1.4**	**2.5**	2.8	1.8	4.4	**8.8**	**6.0**	**13**	2.0	1.3	3.2	**7.1**	**4.8**	**10**	1.5	0.98	2.2	**4.8**	**3.3**	**6.7**
	Switched	0.21	0.11	0.41	0.82	0.38	1.8	**4.0**	**2.5**	**6.3**	3.4	1.6	7.0	**18**	**9.5**	**34**	2.8	1.3	5.8	**14**	**7.2**	**25**	1.3	0.59	2.7	**8.1**	**4.3**	**16**
9V	controls	0.21	0.13	0.35	5.2	2.9	9.1	**20**	**12**	**33**	4.7	2.8	7.6	**23**	**15**	**36**	3.1	1.9	5.2	**13**	**8.6**	**20**	2.1	1.3	3.4	**10**	**6.8**	**15**
	cIM	0.18	0.12	0.29	1.6	0.99	2.6	**7.8**	**5.1**	**12**	1.4	0.94	2.2	**8.7**	**5.9**	**13**	1.3	0.86	2.1	**6.5**	**4.4**	**9.5**	1.1	0.70	1.6	**5.5**	**3.9**	**7.9**
	bIM	0.17	0.11	0.27	1.5	0.95	2.4	**8.6**	**5.7**	**13**	1.6	1.0	2.4	**8.1**	**5.6**	**12**	1.2	0.77	1.9	**6.2**	**4.2**	**9.2**	0.76	0.50	1.2	**4.4**	**3.1**	**6.2**
	Combi	0.22	0.15	0.33	1.7	1.1	2.6	**7.5**	**5.2**	**11**	1.7	1.2	2.5	**7.6**	**5.3**	**11**	1.2	0.84	1.9	**5.7**	**4.0**	**8.0**	0.81	0.56	1.2	**3.6**	**2.7**	**4.9**
	Switched	0.23	0.12	0.46	3.5	1.7	7.0	**15**	**8.0**	**27**	2.7	1.4	5.1	**11**	**5.9**	**19**	1.7	0.86	3.2	**7.9**	**4.5**	**14**	1.1	0.56	2.2	**4.9**	**2.8**	**8.6**
10A	controls	0.70	0.43	1.1	0.97	0.58	1.6	1.2	0.95	1.4	4.6	2.4	8.9	**7.1**	**4.6**	**11**	3.8	2.0	7.5	**5.6**	**3.7**	**8.5**	3.3	1.8	6.1	**4.8**	**3.2**	**7.0**
	cIM	0.59	0.39	0.89	0.64	0.41	1.0	1.0	0.86	1.2	4.6	2.6	8.1	**7.5**	**5.1**	**11**	4.1	2.2	7.4	**6.7**	**4.6**	**9.8**	3.6	2.1	6.2	**5.8**	**4.1**	**8.1**
	bIM	0.45	0.30	0.67	0.52	0.34	0.79	1.1	0.96	1.3	2.7	1.6	4.8	**6.4**	**4.4**	**9.4**	1.9	1.1	3.6	**5.0**	**3.4**	**7.3**	1.7	1.0	2.9	**3.9**	**2.8**	**5.4**
	Combi	0.34	0.24	0.50	0.39	0.27	0.58	1.1	0.95	1.3	2.1	1.2	3.5	**6.3**	**4.4**	**9.0**	1.8	1.1	3.1	**5.0**	**3.6**	**7.0**	1.2	0.76	2.0	**3.5**	**2.6**	**4.8**
	Switched	0.31	0.16	0.57	0.44	0.23	0.83	1.4	1.1	1.8	2.6	1.1	6.2	**9.0**	**5.0**	**16**	1.5	0.64	3.6	**5.2**	**3.0**	**8.9**	0.82	0.34	2.0	**2.9**	**1.7**	**5.1**
11A	controls	0.54	0.32	0.90	0.68	0.38	1.2	1.2	1.0	1.4	4.1	2.4	7.0	**8.0**	**5.3**	**12**	3.6	2.1	6.1	**6.9**	**4.6**	**10**	2.6	1.6	4.3	**4.7**	**3.2**	**6.7**
	cIM	0.48	0.30	0.75	0.51	0.31	0.83	1.0	0.88	1.2	2.9	1.8	4.6	**5.9**	**4.1**	**8.4**	2.4	1.5	3.8	**4.8**	**3.3**	**6.9**	2.0	1.3	3.1	**3.9**	**2.8**	**5.4**

Table S4 Geometric mean concentrations and geometric mean fold rises compared to baseline.

		T0			T2						T4						T6						T12					
			95% CI		GMC	95% CI			95% CI		GMC	95% CI			95% CI		GMC	95% CI			95% CI		GMC	95% CI			95% CI	
**Serotype**	**Group**	**GMC**	Lower	Upper		Lower	Upper	GMFR	Lower	Upper		Lower	Upper	GMFR	Lower	Upper		Lower	Upper	GMFR	Lower	Upper		Lower	Upper	GMFR	Lower	Upper
	bIM	0.49	0.32	0.76	0.56	0.35	0.89	1.1	0.96	1.3	2.6	1.6	4.0	**5.2**	**3.7**	**7.4**	2.3	1.4	3.7	**4.6**	**3.2**	**6.6**	1.5	0.97	2.3	**3.2**	**2.4**	**4.4**
	Combi	0.51	0.34	0.76	0.57	0.37	0.87	1.1	0.97	1.3	2.3	1.5	3.6	**4.7**	**3.4**	**6.5**	2.0	1.3	3.0	**3.7**	**2.7**	**5.1**	1.4	0.95	2.0	**2.8**	**2.1**	**3.7**
	Switched	0.53	0.27	1.0	0.65	0.32	1.3	1.2	0.99	1.5	3.0	1.5	6.0	**6.1**	**3.6**	**10**	3.2	1.6	6.4	**5.0**	**3.0**	**8.4**	1.4	0.71	2.9	**3.2**	**1.9**	**5.5**
12F	controls	0.06	0.04	0.09	0.12	0.07	0.19	1.9	1.4	2.5	0.83	0.43	1.6	**18**	**11**	**30**	0.87	0.44	1.7	**16**	**9.5**	**27**	0.55	0.30	1.0	**9.4**	**5.8**	**15**
	cIM	0.06	0.04	0.09	0.08	0.05	0.12	1.3	1.1	1.7	0.99	0.56	1.7	**16**	**10**	**26**	0.76	0.41	1.4	**14**	**8.5**	**22**	0.68	0.39	1.2	**12**	**7.6**	**18**
	bIM	0.05	0.03	0.07	0.08	0.06	0.12	1.6	1.3	2.0	0.50	0.28	0.87	**9.2**	**5.9**	**14**	0.46	0.25	0.86	**8.5**	**5.3**	**14**	0.28	0.16	0.48	**5.4**	**3.5**	**8.2**
	Combi	0.04	0.03	0.06	0.07	0.05	0.09	1.6	1.3	1.9	0.36	0.21	0.60	**8.6**	**5.7**	**13**	0.28	0.16	0.47	**6.9**	**4.6**	**10**	0.21	0.13	0.34	**5.1**	**3.5**	**7.4**
	Switched	0.04	0.02	0.06	0.09	0.05	0.16	2.4	1.7	3.5	0.86	0.36	2.02	**26**	**13**	**51**	0.57	0.23	1.4	**16**	**8.1**	**31**	0.32	0.13	0.78	**8.5**	**4.2**	**17**
14	controls	0.81	0.45	1.5	8.0	4.4	15	**9.6**	**5.8**	**16**	8.5	4.8	15	**11**	**6.7**	**18**	6.6	3.6	12	**8.2**	**5.1**	**13**	4.9	2.8	8.5	**6.4**	**4.1**	**10.0**
	cIM	1.5	0.88	2.4	9.3	5.6	15	**5.7**	**3.7**	**8.79**	11	6.8	18	**7.9**	**5.2**	**12**	9.9	5.8	17	**6.5**	**4.2**	**9.9**	8.2	5.1	13	**5.3**	**3.6**	**7.8**
	bIM	0.78	0.47	1.3	10	6.2	16	**12**	**8.2**	**19**	8.6	5.3	14	**11**	**7.3**	**17**	7.2	4.2	12	**9.5**	**6.2**	**15**	5.3	3.3	8.5	**6.6**	**4.5**	**9.6**
	Combi	0.86	0.55	1.4	6.6	4.2	10	**7.1**	**4.9**	**10**	6.9	4.4	11	**8.0**	**5.4**	**12**	5.8	3.6	9.3	**6.6**	**4.5**	**9.6**	3.7	2.4	5.7	**4.6**	**3.3**	**6.4**
	Switched	0.64	0.30	1.4	17	8.1	35	**26**	**14**	**49**	16	7.5	33	**23**	**12**	**44**	12	5.3	25	**18**	**9.5**	**32**	6.9	3.2	15	**8.2**	**4.4**	**15**
15B	controls	0.96	0.57	1.6	2.1	1.1	3.8	1.9	1.4	2.6	10	6.0	18	**11**	**7.0**	**18**	9.7	5.6	17	**11**	**6.9**	**16**	6.5	3.9	11	**6.5**	**4.3**	**9.7**
	cIM	0.87	0.56	1.4	1.5	0.89	2.5	1.6	1.2	2.1	6.1	3.8	9.9	**6.9**	**4.6**	**10**	4.8	2.9	7.9	**5.5**	**3.7**	**8.0**	4.5	2.8	7.1	**5.1**	**3.6**	**7.3**
	bIM	0.78	0.50	1.2	1.2	0.71	1.9	1.4	1.1	1.9	5.8	3.6	9.3	**7.2**	**4.8**	**11**	5.0	3.1	8.3	**6.4**	**4.4**	**9.4**	3.3	2.1	5.1	**4.4**	**3.1**	**6.3**
	Combi	0.52	0.35	0.77	0.89	0.57	1.4	1.6	1.3	2.1	4.3	2.7	6.7	**8.0**	**5.5**	**12**	3.4	2.2	5.2	**6.4**	**4.6**	**8.9**	2.3	1.5	3.4	**4.4**	**3.2**	**6.1**
	Switched	0.71	0.36	1.4	1.2	0.57	2.5	1.7	1.1	2.5	6.7	3.2	14	**8.6**	**4.7**	**16**	4.3	2.1	8.7	**6.3**	**3.6**	**11**	2.9	1.4	6.0	**3.6**	**2.0**	**6.5**
17F	controls	0.64	0.35	1.2	0.60	0.31	1.1	0.97	0.75	1.3	12	6.2	23	**17**	**10**	**28**	10	5.2	20	**14**	**8.2**	**23**	6.2	3.3	12	**9.5**	**6.0**	**15**
	cIM	0.35	0.21	0.59	0.44	0.25	0.76	1.1	0.91	1.4	3.1	1.8	5.6	**8.3**	**5.3**	**13**	3.2	1.7	5.9	**7.3**	**4.6**	**12**	2.3	1.3	4.1	**6.2**	**4.1**	**9.5**
	bIM	0.41	0.24	0.67	0.50	0.30	0.85	1.2	0.95	1.5	3.8	2.2	6.6	**9.5**	**6.1**	**15**	4.0	2.2	7.4	**9.4**	**5.9**	**15**	2.4	1.4	4.1	**6.0**	**4.0**	**9.1**
	Combi	0.37	0.23	0.58	0.46	0.28	0.75	1.3	1.0	1.5	2.5	1.5	4.2	**6.6**	**4.3**	**10**	1.9	1.1	3.3	**5.8**	**3.9**	**8.6**	1.4	0.87	2.3	**3.6**	**2.5**	**5.2**
	Switched	0.47	0.21	1.03	0.62	0.28	1.4	1.3	0.96	1.8	5.8	2.5	14	**13**	**6.6**	**26**	4.0	1.7	9.8	**10**	**5.3**	**20**	2.1	0.85	5.2	**4.9**	**2.5**	**9.6**
18C	controls	0.52	0.31	0.88	13	7.1	25	**21**	**13**	**36**	11	6.2	20	**23**	**14**	**36**	7.1	3.9	13	**16**	**10**	**25**	5.8	3.3	10	**11**	**7.2**	**16**
	cIM	0.35	0.22	0.56	4.3	2.5	7.5	**12**	**7.5**	**18**	4.2	2.5	7.1	**12**	**8.0**	**18**	3.6	2.1	6.3	**10**	**6.7**	**15**	2.8	1.7	4.6	**7.1**	**4.9**	**10**
	bIM	0.34	0.22	0.53	4.3	2.6	7.2	**12**	**8.1**	**19**	4.2	2.5	7.0	**12**	**7.9**	**18**	3.0	1.7	5.1	**8.7**	**5.8**	**13**	2.0	1.2	3.2	**5.6**	**3.9**	**8.0**
	Combi	0.38	0.25	0.57	3.8	2.3	6.1	**10**	**7.1**	**15**	3.7	2.3	5.9	**11**	**7.3**	**15**	3.2	2.0	5.2	**8.4**	**5.8**	**12**	2.0	1.3	3.0	**5.4**	**3.9**	**7.4**
	Switched	0.36	0.18	0.72	6.4	2.9	14	**18**	**9.5**	**34**	4.4	2.0	9.5	**12**	**6.6**	**22**	3.4	1.6	7.5	**9.0**	**5.0**	**16**	1.6	0.7	3.5	**4.7**	**2.6**	**8.5**

Table S4 Geometric mean concentrations and geometric mean fold rises compared to baseline.																												
		**T0**			**T2**						**T4**						**T6**						**T12**					
			95% CI		GMC	95% CI			95% CI		GMC	95% CI			95% CI		GMC	95% CI			95% CI		GMC	95% CI			95% CI	
**Serotype**	**Group**	**GMC**	Lower	Upper		Lower	Upper	GMFR	Lower	Upper		Lower	Upper	GMFR	Lower	Upper		Lower	Upper	GMFR	Lower	Upper		Lower	Upper	GMFR	Lower	Upper
19A	controls	2.7	1.6	4.3	29	17	47	10	6.4	16	25	15	40	9.2	6.0	14	18	11	29	6.5	4.3	9.9	13	8.2	20	4.7	3.2	7.0
	cIM	0.62	0.40	0.94	6.0	3.9	9.1	8.8	5.9	13	4.7	3.1	7.2	7.7	5.3	11	4.0	2.6	6.3	6.8	4.6	9.9	3.3	2.2	5.0	5.0	3.5	7.1
	bIM	0.84	0.56	1.3	6.1	4.1	9.2	7.6	5.2	11	6.2	4.1	9.3	7.4	5.1	11	4.6	3.0	7.2	5.2	3.6	7.6	2.9	2.0	4.4	3.6	2.5	5.0
	Combi	0.99	0.68	1.4	6.7	4.6	9.8	6.9	4.9	9.9	6.4	4.4	9.4	6.3	4.5	8.9	5.1	3.4	7.4	5.2	3.8	7.3	3.6	2.5	5.1	3.5	2.6	4.8
	Switched	0.80	0.43	1.5	9.5	5.1	18	12	6.6	21	9.8	5.2	18	11	6.4	20	6.9	3.7	13	8.1	4.7	14	4.3	2.3	8.3	4.9	2.8	8.6
19F	controls	0.93	0.56	1.6	10	5.9	18	10	6.2	16	10	6.3	17	11	7.1	17	6.8	4.2	11	7.9	5.1	12	5.4	3.4	8.7	5.5	3.7	8.1
	cIM	0.58	0.37	0.90	3.8	2.4	6.1	6.6	4.4	9.9	4.1	2.7	6.3	7.3	5.0	11	3.4	2.2	5.3	6.1	4.2	9.0	2.8	1.8	4.2	4.5	3.2	6.5
	bIM	0.60	0.39	0.93	4.3	2.7	6.6	7.5	5.1	11	4.4	2.9	6.8	7.8	5.3	11	3.4	2.2	5.2	6.5	4.4	9.5	2.2	1.5	3.4	4.1	2.9	5.7
	Combi	0.61	0.41	0.90	4.4	2.9	6.6	7.3	5.2	10	4.5	3.1	6.7	7.5	5.3	11	3.5	2.4	5.1	5.6	4.0	7.9	2.2	1.5	3.2	3.6	2.7	4.9
	Switched	0.49	0.25	0.95	6.9	3.5	14	14	7.9	25	6.1	3.2	12	13	7.3	23	4.5	2.4	8.5	9.5	5.4	17	2.7	1.4	5.4	5.2	2.9	9.2
20	controls	1.4	0.85	2.2	1.6	1.0	2.7	1.2	0.95	1.4	9.5	5.5	17	7.1	4.5	11	7.2	4.1	13	5.3	3.5	8.0	6.3	3.6	11	4.6	3.1	6.7
	cIM	0.64	0.42	0.97	0.78	0.51	1.2	1.1	0.97	1.4	4.4	2.7	7.1	7.3	5.0	11	3.8	2.3	6.4	6.1	4.2	8.9	3.5	2.1	5.6	5.3	3.8	7.4
	bIM	1.3	0.87	2.0	1.5	0.99	2.2	1.2	0.99	1.4	8.0	5.0	13	5.6	3.8	8.2	7.1	4.3	12	4.9	3.4	7.2	4.8	3.0	7.7	3.6	2.6	5.1
	Combi	1.1	0.79	1.7	1.4	0.95	2.0	1.3	1.1	1.5	7.2	4.6	11	5.9	4.2	8.4	5.0	3.2	7.8	4.5	3.2	6.2	3.9	2.6	6.0	3.4	2.5	4.5
	Switched	0.68	0.36	1.3	1.1	0.57	1.9	1.5	1.2	2.0	4.1	2.0	8.4	6.2	3.5	11	4.9	2.3	10	6.2	3.6	11	2.1	0.94	4.6	3.3	1.9	5.6
22F	controls	0.17	0.10	0.29	0.22	0.13	0.39	1.2	0.90	1.6	5.1	2.8	9.2	28	16	48	3.7	2.0	6.8	20	12	35	2.2	1.2	3.8	13	7.9	22
	cIM	0.14	0.09	0.22	0.14	0.08	0.22	1.1	0.87	1.4	2.2	1.3	3.8	16	9.9	26	2.0	1.1	3.4	13	7.8	21	1.5	0.90	2.5	10	6.5	16
	bIM	0.15	0.09	0.22	0.18	0.12	0.29	1.2	0.99	1.6	1.8	1.1	2.9	12	7.8	20	1.7	0.98	3.0	11	6.9	19	1.1	0.67	1.8	6.7	4.3	10
	Combi	0.15	0.10	0.22	0.16	0.11	0.24	1.1	0.86	1.3	1.4	0.90	2.3	9.2	5.9	14	1.2	0.72	1.9	7.9	5.1	12	0.76	0.49	1.2	5.0	3.4	7.4
	Switched	0.12	0.06	0.24	0.18	0.09	0.36	1.5	1.0	2.1	2.5	1.1	5.4	21	10	43	1.7	0.76	3.7	14	6.8	28	0.92	0.41	2.1	7.1	3.4	15
23F	controls	0.34	0.19	0.60	11	5.6	21	31	18	53	10.0	5.4	18	27	17	43	6.6	3.6	12	22	13	35	4.5	2.5	8.2	14	8.7	22
	cIM	0.18	0.11	0.30	2.8	1.6	5.0	13	8.5	21	2.7	1.6	4.5	16	10	24	1.7	0.96	2.9	11	6.9	17	1.6	0.96	2.8	8.6	5.7	13
	bIM	0.15	0.09	0.25	2.9	1.7	4.9	19	12	29	2.5	1.5	4.2	17	11	26	1.9	1.1	3.2	13	8.6	21	1.3	0.76	2.1	8.1	5.4	12
	Combi	0.21	0.13	0.33	2.5	1.5	4.1	13	8.4	19	2.4	1.5	3.9	11	7.6	16	1.9	1.2	3.1	8.0	5.4	12	1.1	0.72	1.8	5.5	3.8	7.9
	Switched	0.22	0.10	0.48	8.6	3.8	19	39	20	75	6.1	2.8	13	27	14	50	4.7	2.1	10	22	12	41	2.3	0.99	5.5	11	5.5	21
33F	controls	1.1	0.67	1.8	1.1	0.66	1.9	1.1	0.90	1.3	12	6.6	22	12	7.2	19	11	6.3	20	9.6	6.1	15	8.5	5.0	15	7.8	5.0	12
	cIM	0.90	0.59	1.4	0.95	0.60	1.5	1.0	0.86	1.2	9.0	5.4	15	10	6.8	16	9.7	5.7	16	9.5	6.3	14	7.4	4.6	12	7.2	4.8	11
	bIM	0.80	0.53	1.2	0.84	0.54	1.3	1.0	0.91	1.2	6.1	3.7	10	8.1	5.3	12	5.7	3.4	9.7	6.7	4.5	10	3.8	2.4	6.1	4.5	3.1	6.6
	Combi	0.62	0.43	0.91	0.72	0.48	1.1	1.1	0.97	1.3	4.8	3.0	7.6	7.7	5.3	11	4.0	2.5	6.4	6.8	4.7	9.7	2.9	1.9	4.5	4.8	3.4	6.7

Table S4 Geometric mean concentrations and geometric mean fold rises compared to baseline.																												
		T0			T2						T4						T6						T12					
			95% CI			95% CI			95% CI			95% CI			95% CI			95% CI			95% CI			95% CI			95% CI	
Serotype	Group	GMC	Lower	Upper	GMC	Lower	Upper	GMFR	Lower	Upper	GMC	Lower	Upper	GMFR	Lower	Upper	GMC	Lower	Upper	GMFR	Lower	Upper	GMC	Lower	Upper	GMFR	Lower	Upper
	Switched	0.52	0.27	0.98	0.71	0.37	1.4	1.4	1.1	1.7	9.2	4.3	20	**18**	**9.6**	**34**	7.1	3.4	15	**14**	**7.7**	**25**	4.8	2.2	10	**7.4**	**3.9**	**14**
12A	controls	1.17	0.70	1.95	2.1	1.3	3.5	**1.6**	**1.3**	**1.9**	2.6	1.5	4.3	**2.2**	**1.6**	**2.9**	2.6	1.4	4.5	**2.0**	**1.4**	**2.7**	1.9	1.1	3.3	**1.7**	**1.3**	**2.3**
	cIM	0.67	0.43	1.05	0.83	0.54	1.3	1.2	0.98	1.4	1.5	0.94	2.3	**2.2**	**1.7**	**2.8**	1.5	0.87	2.4	**2.4**	**1.8**	**3.1**	1.32	0.82	2.1	**2.0**	**1.5**	**2.5**
	bIM	0.64	0.41	0.98	0.81	0.54	1.2	**1.3**	**1.1**	**1.5**	1.3	0.83	2.0	**2.0**	**1.5**	**2.5**	1.2	0.70	2.0	**1.8**	**1.4**	**2.4**	1.03	0.65	1.6	**1.7**	**1.3**	**2.2**
	Combi	0.47	0.32	0.70	0.61	0.41	0.89	**1.4**	**1.2**	**1.6**	0.88	0.59	1.3	**1.9**	**1.5**	**2.3**	0.83	0.53	1.3	**1.7**	**1.3**	**2.1**	0.70	0.46	1.1	**1.5**	**1.2**	**1.8**
	Switched	0.57	0.29	1.11	0.83	0.45	1.56	**1.5**	**1.2**	**1.9**	0.92	0.47	1.8	**1.6**	**1.1**	**2.3**	0.91	0.43	1.9	1.4	0.96	2.2	0.74	0.34	1.6	1.2	0.8	1.8
45	controls	5.3	3.5	8.0	7.1	4.5	11	1.16	0.98	1.4	6.0	3.8	9.3	1.2	1.0	1.4	5.5	3.5	8.6	1.1	0.89	1.3	6.8	4.4	11	**1.3**	**1.1**	**1.6**
	cIM	3.7	2.6	5.3	4.9	3.3	7.2	1.2	1.0	1.4	4.5	3.1	6.6	**1.2**	**1.1**	**1.4**	4.6	3.1	6.9	**1.2**	**1.1**	**1.5**	4.6	3.1	6.8	1.2	1.0	1.4
	bIM	3.7	2.6	5.3	4.3	3.0	6.3	1.2	1.0	1.3	5.5	3.7	8.0	**1.2**	**1.1**	**1.4**	4.4	2.9	6.6	**1.2**	**1.0**	**1.4**	4.5	3.0	6.5	**1.3**	**1.1**	**1.5**
	Combi	2.9	2.1	4.0	3.0	2.1	4.2	1.0	0.92	1.2	3.3	2.3	4.7	1.1	0.96	1.2	3.1	2.2	4.5	1.1	0.92	1.2	2.9	2.1	4.1	1.0	0.9	1.2
	Switched	2.8	1.6	4.8	2.7	1.5	4.8	0.97	0.79	1.2	2.7	1.5	4.8	0.96	0.77	1.2	3.1	1.7	5.5	1.00	0.79	1.3	2.9	1.6	5.5	0.9	0.6	1.2

T= time point, GMC = geometric mean concentration, GMFR = geometric mean fold rise, cIM= conventional immunomodulator, bIM = biological immunomodulator, combi = combination therapy

* 12A and 45 serotypes are in AU/ml

Blue = PCV13 serotypes, white = PPSV23-Non-PCV13 serotypes, green= non-vaccine serotypes

Bold highlights statistically significant geometric mean fold rise from baseline

Table S5, Overview of serious adverse events

Age	Sex	Relatedness to study	Months after enrollment	Underlying disease	Description of event
**29**	male	Unrelated	11	Neurological disease	Herpes zoster infection requiring hospital admission and treatment
**22**	male	Unrelated	5	M Crohn	Hospital admission for abdominal pain (stenosis)
**22**	male	Unrelated	4	M Crohn	Hospital admission for abdominal pain (stenosis)
**58**	female	Unrelated	4	Renal transplant recipient	Urinary tract infection
**58**	female	Unrelated	2	Renal transplant recipient	Asthma renale

**58**	female	Unrelated	9	Renal transplant recipient	Urinary tract infection and CMV-colitis, transplant failure (chronic rejection)
**58**	male	Unlikely	0	Spondylitis ankylopoietica	Herpes zoster infection requiring hospital admission and treatment
**54**	male	Unrelated	2	Colitis ulcerosa	Urinary tract infection and bowel obstruction requiring admission
**68**	female	Unrelated	2	Rheumatoid arthritis	Pancreatitis due to methotrexate use
**49**	male	Unrelated	5	Renal transplant recipient	Multi-organ failure due to bloodstream infection, patient died
**36**	male	Unrelated	2	M Crohn	Severe anemia due to purinethol requiring hospital admission
**50**	male	Unrelated	8	Renal transplant recipient	1. Severe COVID-19 2. AKI and transplant failure due to 1.
**70**	female	Unrelated	9	Colitis ulcerosa	Intestinal bleeding after biopsy
**64**	female	Unrelated	5	Renal transplant recipient	Hypoglycemia requiring hospital admission
**26**	male	Unlikely	11	Renal transplant recipient	Combined graft rejection requiring hospital admission and treatment
**58**	female	Unrelated	1	Asthma	Severe COVID-19
**53**	male	Unlikely	7	Renal transplant recipient	Humoral graft rejection requiring hospital admission and treatment
